# Direct Measurements of Local Coupling between Myosin Molecules Are Consistent with a Model of Muscle Activation

**DOI:** 10.1371/journal.pcbi.1004599

**Published:** 2015-11-04

**Authors:** Sam Walcott, Neil M. Kad

**Affiliations:** 1 Mathematics, University of California at Davis, Davis, California, United States of America; 2 School of Biosciences, University of Kent, Canterbury, Kent, United Kingdom; University of California San Diego, UNITED STATES

## Abstract

Muscle contracts due to ATP-dependent interactions of myosin motors with thin filaments composed of the proteins actin, troponin, and tropomyosin. Contraction is initiated when calcium binds to troponin, which changes conformation and displaces tropomyosin, a filamentous protein that wraps around the actin filament, thereby exposing myosin binding sites on actin. Myosin motors interact with each other indirectly via tropomyosin, since myosin binding to actin locally displaces tropomyosin and thereby facilitates binding of nearby myosin. Defining and modeling this local coupling between myosin motors is an open problem in muscle modeling and, more broadly, a requirement to understanding the connection between muscle contraction at the molecular and macro scale. It is challenging to directly observe this coupling, and such measurements have only recently been made. Analysis of these data suggests that two myosin heads are required to activate the thin filament. This result contrasts with a theoretical model, which reproduces several indirect measurements of coupling between myosin, that assumes a single myosin head can activate the thin filament. To understand this apparent discrepancy, we incorporated the model into stochastic simulations of the experiments, which generated simulated data that were then analyzed identically to the experimental measurements. By varying a single parameter, good agreement between simulation and experiment was established. The conclusion that two myosin molecules are required to activate the thin filament arises from an assumption, made during data analysis, that the intensity of the fluorescent tags attached to myosin varies depending on experimental condition. We provide an alternative explanation that reconciles theory and experiment without assuming that the intensity of the fluorescent tags varies.

## Author Summary

Despite decades of study, there is no clear connection between muscle contraction at the molecular and the macroscopic scale. For example, we cannot yet predict how a genetic defect in a muscle protein will result in a physiological change in the heart. This multi-scale understanding is difficult, in part, because molecules cooperate during muscle contraction; that is, one molecule’s behavior is influenced by the behavior of its neighbors. It is difficult to make direct measurements from such coupled molecular systems and also difficult to describe them quantitatively. Despite these obstacles, we recently published experimental measurements and theoretical models of this coupling, but there were apparent discrepancies between the two. Here, we use detailed computer simulations of these experiments to show that, in fact, the measurements agree with the model to a remarkable extent. This agreement suggests that the model captures the essential molecular events that underlie the coupling between muscle molecules. This removes a major obstacle to a multi-scale understanding of muscle contraction and, while more work is necessary, suggests that a connection between the molecular and macroscopic scale is within reach.

## Introduction

**Fig 1:**
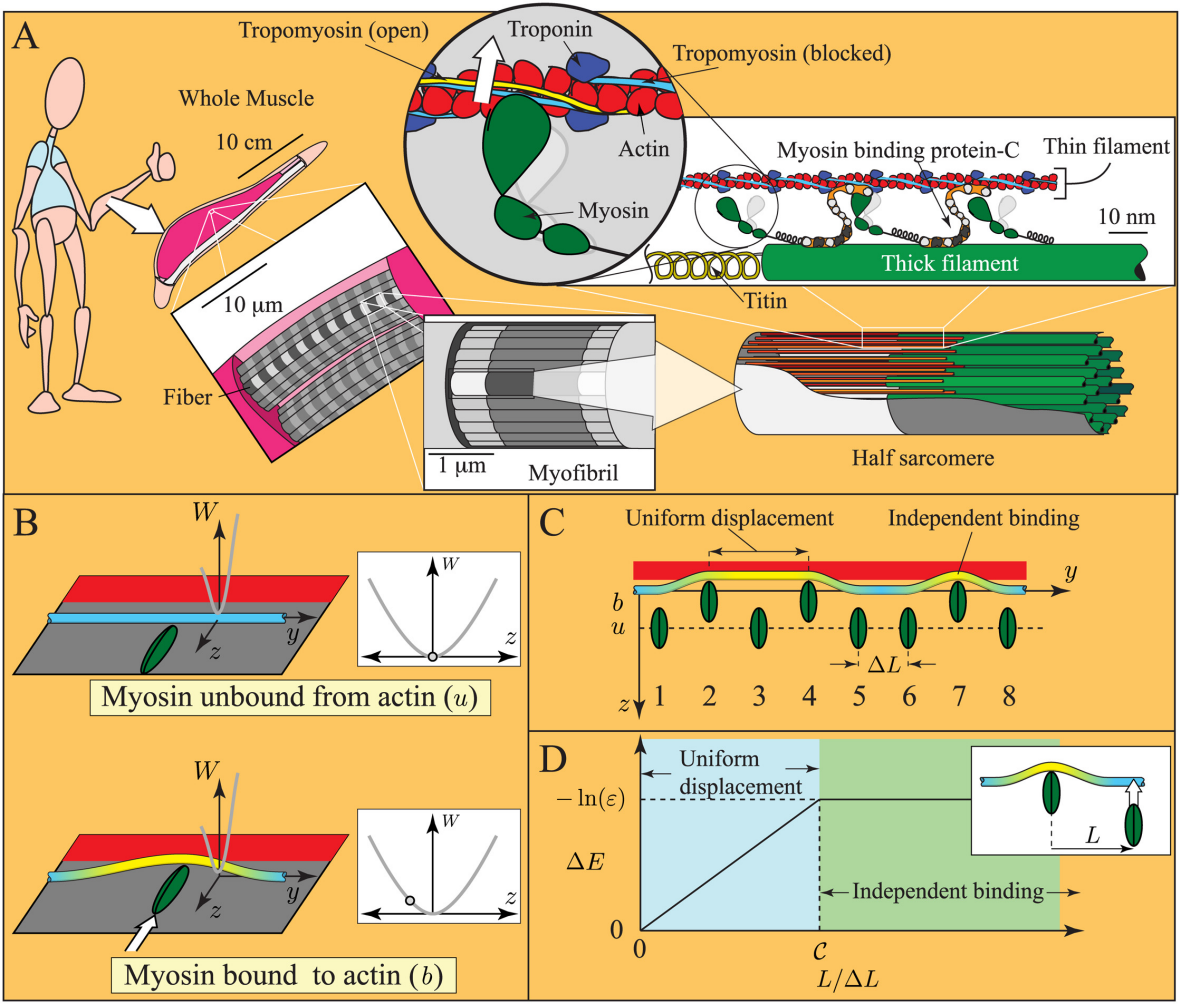
Cartoons of muscle and the model of local coupling. A. Size scales in muscle. Muscle contracts due to the formation of transient links between thick filaments (made primarily of the protein myosin) and thin filaments (made of the proteins actin, troponin and tropomyosin). Contraction is regulated by troponin and tropomyosin. After binding calcium, troponin moves tropomyosin from a position where myosin binding to actin is obstructed (blue, blocked position) toward a position where myosin binding is unhindered (yellow, open position). B. Assumptions of the continuous flexible chain model [15–17]. Troponin-tropomyosin (Tn-Tm) is a slender, infinite, linearly elastic beam constrained to a plane and in a potential well, *W*(*z*). Myosin binding to actin induces a displacement of the Tn-Tm beam into the open position (yellow), and locally deforms it. C. Simplifying assumptions of the model. When nearby myosin molecules bind to actin (molecules 2 and 4), they uniformly displace the intervening Tn-Tm beam into the open position. When distant myosin molecules bind to actin (molecules 4 and 7), they each induce independent deformations of the Tn-Tm beam. D. Energy change of the thin filament (Δ*E*) due to myosin binding, as a function of the distance to a bound myosin (*L*). When *L* is small, Tn-Tm is displaced uniformly, and Δ*E* increases linearly with *L* (blue region); when *L* is large, each myosin displaces Tn-Tm independently, and Δ*E* is independent of *L* (green region). If the transition between these regimes is abrupt, the curve is defined by two non-dimensional parameters, C and *ε*. Note: in the presence of calcium, the Tn-Tm beam moves into the closed state (not pictured), which changes *ε*, but not C.

Muscle contraction underlies most voluntary and involuntary motion of multicellular animals. The last 100 years have revolutionized our understanding of this process. Advances in light microscopy in the early 20th century culminated in the sliding filament theory, the discovery that muscle contracts via the relative sliding of two sets of protein filaments (Fig 1A) [1, 2]. To achieve this motion, it was hypothesized that myosin molecules in thick filaments form transient bonds with specific binding sites on actin in thin filaments [3]. Subsequent experiments characterized the biochemical reactions between myosin and actin, culminating in the determination of how these reactions couple to the hydrolysis of ATP [4]. More recently, single molecule techniques have allowed direct observation of myosin binding to actin [5] and measurement of the effects of external force on this chemical interaction [6, 7]. However, despite this wealth of knowledge, a clear understanding of the connection between muscle contraction at the molecular and cell or organ scale remains unclear. Besides integrating a century of research on muscle physiology, such an understanding might allow, for example, novel treatments of genetic heart disease [8].

Connecting the molecular and macro scales of muscle contraction is a complex problem. Forming this connection requires 1) understanding how an isolated myosin molecule interacts with a thin filament; 2) understanding how this interaction changes when multiple myosin molecules work together; 3) understanding how the collective behavior of multiple myosin motors changes upon the addition of accessory proteins like titin and myosin binding protein-C (MyBP-C); and 4) understanding how collections of myosin with accessory proteins work together when arranged as sarcomeres in series, myofibrils in parallel and so on up to whole muscle (Fig 1A). While recent single molecule experiments have given a relatively clear picture of how isolated myosin molecules interact with actin filaments e.g. [5–7], the remaining three ingredients of a molecular to macro scale understanding remain much less clear.

Thus, one obstacle to connecting muscle at the molecular and macro scale is that myosin motors interact with each other via a common actin filament. This coupling occurs in two ways. The first, which we call mechanochemical coupling, occurs because myosin motors apply forces on each other when they bind to the same actin filament. These forces can accelerate chemical reaction rates, and lead to emergent ensemble behavior [9, 10]. If the actin filament is relatively stiff, then this coupling affects every molecule equally—that is, it doesn’t matter where on the actin filament the bound myosin molecules are located.

The second type of coupling, herein termed local coupling, occurs when a myosin molecule binds to the actin filament and affects the reaction rates of nearby myosin molecules, but has no effect on distant myosin. Local coupling happens when myosin binds to actin at low calcium in the presence of the regulatory proteins troponin and tropomyosin. Under these conditions, the protein tropomyosin, which wraps around the actin filament, sterically hinders myosin binding by covering the binding sites on actin (Fig 1A) [11]. If a myosin molecule then binds, it deforms tropomyosin. This deformation, being local, makes it easier for neighboring myosin molecules to bind to actin, but has no effect on distant myosin molecules (Fig 1B–1D) [12–17]. Direct observation of either type of coupling presents a major challenge, since traditional single molecule techniques cannot be used. With the lack of direct measurements, mathematical models can be used to interpret indirect observations. However, it is unclear whether mechanochemical and/or local coupling can be captured by current muscle models, since many muscle models incorporate the assumption that molecules act independently.

Mathematical modeling has played an important role in our understanding of the connection between muscle contraction at the micro- and macro-scale. Shortly after the sliding filament and cross-bridge theories of muscle contraction were proposed [1, 2], they were implemented in a mathematical model [3]. Since then, as biochemical and biophysical experiments have uncovered more about the molecular scale, increasingly detailed and increasingly accurate mathematical muscle models have been developed [18–30]. The majority of these models build on the ideas and assumptions of Huxley’s original model [3]. One of these assumptions is that each myosin molecule acts independently of neighboring myosin molecules, the independent force generator assumption [31].

One might expect that any type of coupling between myosin molecules in an ensemble would violate the independent force generator assumption. However, mechanochemical coupling does not. This is because, assuming that the thin and thick filaments are stiff, mechanochemical coupling affects each actin-bound myosin molecule equally, regardless of its position on actin. Thus, the future behavior of each myosin molecule in an ensemble can be determined from the average properties of the ensemble, an alternate statement of the independent force generator assumption [10].

In contrast, local coupling does violate the independent force generator assumption [32]. This is because the future behavior of a myosin ensemble cannot be predicted simply by average ensemble properties, but also depends on the spatial distribution of those properties. For example, two ensembles with, say, 25% of the myosin molecules bound to actin will behave differently if all of the bound molecules are tightly clustered together or if the bound molecules are distributed evenly [13]. Thus, muscle models that incorporate Huxley’s independent force generator assumption cannot capture aspects of muscle activation where local coupling plays an important role.

Since local coupling violates the independent force generator assumption, modeling locally coupled myosin ensembles is challenging. Some of the earliest studies [13] recognized that local coupling occurs in some physical models. In particular, the 1D Ising model considers the behavior of an ensemble of molecules, each of whom is coupled to its nearest neighbor. For this model, analytic expressions can be derived for the equilibrium distribution of the ensemble [13, 33]. But this approach is also limited, in that it is unclear how to model long-distance coupling or non-equilibrium effects, both of which are of central interest in muscle modeling.

An alternate approach is to explicitly model each molecule in a myosin ensemble [14, 34–40]. To capture stochastic effects, Monte-Carlo methods are used [41]. One advantage of this approach is that, since each molecule is considered explicitly, local coupling can be easily included. Thus, effects like filament elasticity, which can contribute to local coupling, can be incorporated into models [35, 37]. These models require significant computational expense, and so have only recently become tractable. But, even with modern computational power, optimizing fits of such models to data is difficult. As a result, most muscle models that include local coupling cannot describe a large suite of experiments.

With the aim of developing a muscle model that describes the largest number of experimental results, a model has recently been proposed that includes both mechanochemical and local coupling in a realistic way and yet can be implemented with minimal computational expense [32, 42, 43]. This model describes a series of experiments that indirectly measure local coupling between myosin molecules. In particular, the local coupling model (summarized in more detail in the Methods section), which has only two parameters, when incorporated into a model of mechanochemical coupling [10], describes measurements of in vitro motility of thin filaments at low ATP [14, 42], at more physiological ATP and in the presence of MyBP-C [43–45] and at physiological ATP and variable calcium [14, 32, 46]. Importantly, the same model with consistent parameters describes these in vitro motility experiments and then reasonably predicts fiber level experiments [32, 47–51]. It is therefore plausible that this model describes how myosin motors work together, and how that interaction changes with the addition of the accessory protein MyBP-C. Given that these are requirements to a multi-scale understanding of muscle contraction, the model represents progress in that direction. However, as yet, the connection of this local coupling model to the molecular scale is speculative, as the model has not been compared to direct molecular-scale measurements.

Recently, the first direct observations of myosin binding to thin filaments were made across a range of activating conditions [52]. Analysis of these data, which clearly demonstrate local coupling, revealed a discrepancy between experiment and theory: the experimental data point towards two heads minimally being required to activate the thin filament whereas the model predicts only one head is required. To understand this discrepancy, we simulated the experimental data using a stochastic method and analyzed the outputs using the same algorithms used for the experimental data. Remarkably, by assuming that fluorescently-tagged myosin emits a constant amount of light per unit time, we found that the model is consistent with the experimental measurements. This assumption, for which we find indirect support, contrasts with an assumption made by Desai et al. [52] that emission depends on experimental condition. Thus, here we propose an alternative scenario where a single myosin head is sufficient to activate a thin filament, thereby reconciling experimental data and theoretical predictions.

## Methods

We adapted a local coupling model [32, 42, 43] of myosin’s interaction with thin filaments to simulate recent single molecule experiments [52]. Here, we provide a brief description of the model, the experiments, and the simulations.

### Model

To describe the local coupling between myosin molecules, we use a simplified mechanochemical model of the thin filament, based on the continuous flexible chain model [15–17]. In this model, troponin/tropomyosin is modeled as a continuous elastic beam, which contrasts with more structurally detailed models of local coupling that explicitly model each troponin and tropomyosin molecule, e.g. [36, 37]. Although this simplifying assumption makes some effects more difficult to include (e.g. potential variations in troponin density [37]), one advantage of this approach is that model parameters are related to physical properties of the system.

The local coupling model used here starts with three assumptions based on the continuous flexible chain model [15–17] 1. troponin/tropomyosin (Tn-Tm) is a slender, infinite, linear-elastic beam constrained to a plane; 2. the interaction of Tn-Tm with the actin filament, described by potential energy density, *W*(*z*), varies azimuthally along actin but does not vary (or the variations can be averaged out) longitudinally along actin; 3. myosin binding induces a local displacement of the Tn-Tm beam. These assumptions are shown in Fig 1B.

A few simplifications differentiate this model from the continuous flexible chain model [42]. The first simplification is based on the observation that the energy of the Tn-Tm beam (*E*) contains two quantities: an elastic component that is minimized when the beam is straight, and a potential energy component that depends on the details of *W*(*z*). Importantly, the elastic component dominates over short distances, while the potential energy component dominates over long distances. Therefore, when two nearby myosin molecules bind to actin, the Tn-Tm beam between the two myosin remains relatively straight, minimizing elastic energy; conversely, when two distant myosin molecules bind to actin, they each induce independent deformations, minimizing potential energy (Fig 1C).

These ideas lead to specific predictions of the energy change of the actin-Tn-Tm system when myosin binds to actin (Δ*E*). Consider, for example, the energy change that occurs when a myosin molecule binds to actin when another myosin molecule, a distance *L* away, has previously bound to actin. If *L* is small, the Tn-Tm beam should be displaced uniformly, minimizing elastic energy. And, since *W*(*z*) does not vary longitudinally along actin, the energy change should increase linearly with *L*. Conversely, if *L* is large, then myosin binding should induce an independent deformation in the Tn-Tm beam, minimizing potential energy, and the energy change should not depend on *L* (see Fig 1D). It is convenient to assume that the transition between these two regimes is abrupt, and occurs at some critical separation *L* = *L*_*C*_. Then, the final curve is defined by two parameters, C and *ε*. The first is the critical length scaled by the separation between myosin molecules, $C=L_{C}/\Delta L$. The second is the reduction in attachment rate due to the regulatory proteins—i.e. if myosin binds to unregulated actin at a rate *k* ^0^, then myosin binds to regulated actin at a rate *εk* ^0^ when *L* ≫ *L*_*C*_ (see Fig 1D).

These results are independent of the details of Tn-Tm’s interaction with actin (i.e. the exact shape of *W*(*z*) [42]). These ideas define any pair-wise interaction between myosin molecules, and therefore we can calculate the energy change that would occur with the binding of any myosin molecule. Assuming that attachment rate depends exponentially on this energy, we can then incorporate regulation into cross-bridge muscle models [10].

More specifically, given a cross-bridge muscle model, regulation is incorporated by modifying the attachment rate and leaving all other rate constants unchanged. Thus, if myosin binds to unregulated actin at a rate *k* ^0^ in a particular model, and supposing that a given molecule’s *i* ^th^ neighbor to the left and *j* ^th^ neighbor to the right are bound to actin, then that given molecule has an attachment rate *k* from the following equation [42]

$$k\left(i,j,C,\varepsilon \right)=\{\begin{array}{ccc} \varepsilon k^{0} & : & i,j\geq C\\ \varepsilon^{i/C}k^{0} & : & i<C,j\geq C\\ \varepsilon^{j/C}k^{0} & : & i\geq C,j<C\\ \varepsilon^{(i+j-C)/C}k^{0} & : & i,j<C,i+j>C\\ k^{0} & : & i+j\leq C \end{array}$$ (1)

As this attachment rate depends on the state of nearby molecules, it introduces local coupling to the model.

Differential equation methods can be used to simulate the behavior of myosin ensembles with both this kind of local coupling and also mechanochemical coupling [32, 42]. These differential equation methods are much more computationally efficient than Monte-Carlo methods, allowing optimization of fits and parameter estimation. Thus, from fits to in vitro motility data, we can obtain values for the parameters C=11 and *ε* = 0.003 at pCa 8 [42], where pCa is the negative log of the calcium concentration. With these parameters, the model fits motility experiments in the presence of MyBP-C, suggesting that MyBP-C both activates the thin filament and specifically binds to actin, thereby competing with myosin and creating a viscous drag [43]. By assuming that calcium only affects *ε*, and that C remains constant, force-pCa experiments from muscle fibers and fiber twitch experiments can be reproduced and then motility-pCa curves and twitch summation experiments are successfully predicted [32]. The reasonable agreement between model and experiments provides support for its assumptions.

If this model is correct, then at low calcium, the binding of one myosin molecule can locally activate the thin filament. Desai et al. [52] interpret their experiments to support the view that at least two myosin molecules are necessary to activate the thin filament, contradicting the model. To understand this difference we used stochastic methods to simulate the experimental observations.

### Experiments

**Fig 2:**
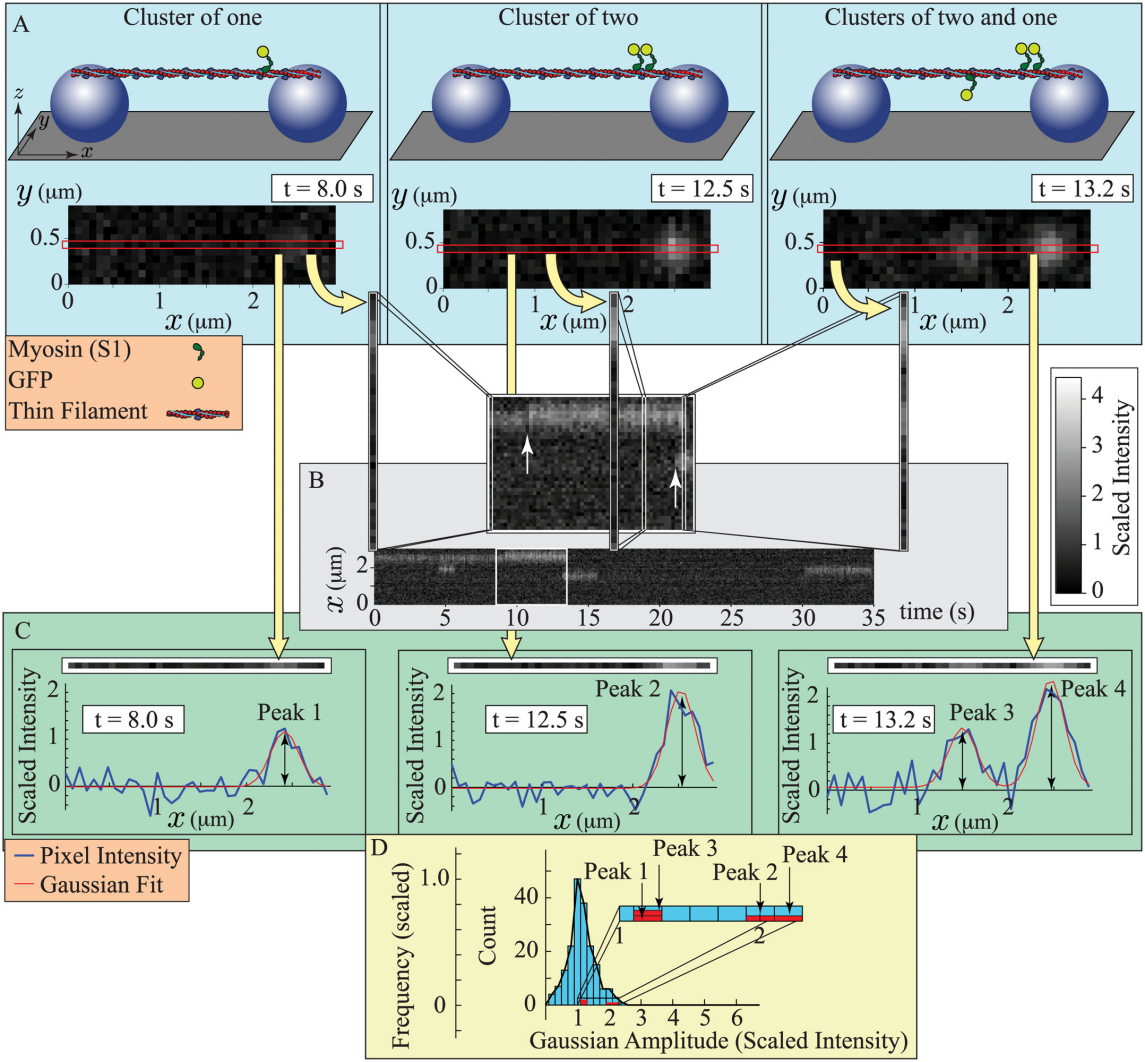
Cartoons describing the experiments and data analysis of Desai et al. [52]. A. Experimental set up and raw images for three different conditions: 1. (left) a single myosin bound to the thin filament; 2. (middle) two nearby myosin molecules bound to the thin filament; and 3. (right) three myosin molecules bound to the thin filament, two of which are close together. (Top) A thin filament is suspended between two silica beads (purple spheres) on a glass surface (gray). Variable concentrations of myosin head domains (S1), labeled with a fluorescent tag (GFP), are added to a solution that also contains variable concentrations of ATP and calcium. (Bottom) A camera records the fluorescence. Bound GFP-tagged S1s (GFP-S1s) appear as diffraction limited spots. In each simulated image, single GFP-S1s make a faint spot, while two nearby GFP-S1s make a brighter spot. The red box indicates the position of the thin filament. B. Construction of a kymograph. For each frame of a recorded movie, the pixels along the thin filament are isolated and stacked together, in chronological order, to make a 2-dimensional image. When GFP-S1s bind to the thin filament (inset, white arrows), they either increase the intensity of an existing spot if they bind near a bound GFP-S1 (left arrow), or they create a new spot (right arrow). C. Fitting the data with Gaussians. For a frame of a recorded movie, the pixels along the thin filament are isolated (top), and fluorescence is plotted as a function of position (bottom). Custom code [52] is used to fit these plots with Gaussians of constant standard deviation, but variable amplitude. Fluorescent intensity as a function of position, and the best-fit Gaussian(s), are shown for each of the different conditions in (A). Two nearby bound GFP-S1s (Peaks 2 and 4) are fit by a Gaussian with roughly twice the amplitude of the single bound GFP-S1 (Peaks 1 and 3). D. Construction of a histogram. For an entire movie, the amplitudes of the best-fit Gaussians of each individual frame are displayed as a histogram. The individual frames shown in (C) generate four amplitudes (red). The histogram is scaled so that the peak has an amplitude of 1. Note: scaled intensity, defined in the text, gives a single GFP-S1 an intensity of 1.

In the previously published experiments, the head domain of myosin (S1) was directly observed binding to thin filaments (actin with troponin and tropomyosin) [52]. The experiment starts by using fluid flow to suspend a thin filament between two silica beads affixed to a glass surface (Fig 2A). Then, a solution is added that includes variable concentrations of 1) GFP-labeled S1 domains of myosin (GFP-S1); 2) ATP; and 3) calcium. The thin filaments are then imaged using Oblique Angle Fluorescence microscopy to detect fluorescent GFP-S1s bound to the thin filament.

With appropriate concentrations of GFP-S1, ATP and calcium, individual fluorescent spots are observed (Fig 2A). These spots can vary in brightness, indicating the binding of multiple GFP-S1s, can appear/disappear or can randomly diffuse along the thin filament. Movies of these spots were visualized in two ways, as kymographs (Fig 2B) and as histograms (Fig 2D).

To generate both a kymograph and a histogram from a movie, Desai et al. [52] performed the following steps. Pixels lying along the thin filament were isolated (Fig 2A, red box). For the kymograph, the intensity along this line of pixels was plotted for each frame of the movie, generating a 2D image with position in the ordinate and time on the abscissa (Fig 2B). Any GFP-S1 interactions with the thin filament appear as streaks that start when the GFP-S1 binds and end when the GFP-S1 detaches.

For a histogram, the intensity of the line of pixels was plotted as a function of pixel position (Fig 2C). These data were then fit with custom code [52] that determines the best-fit Gaussians of fixed standard deviation, but variable amplitude. The amplitudes of these best-fit Gaussians were determined for every frame of the movie and then plotted as a histogram (Fig 2D). As concentrations of GFP-S1, ATP and calcium were varied, clear differences are observed in both the kymographs and the histograms [52].

### Modeling the experiments

**Fig 3:**
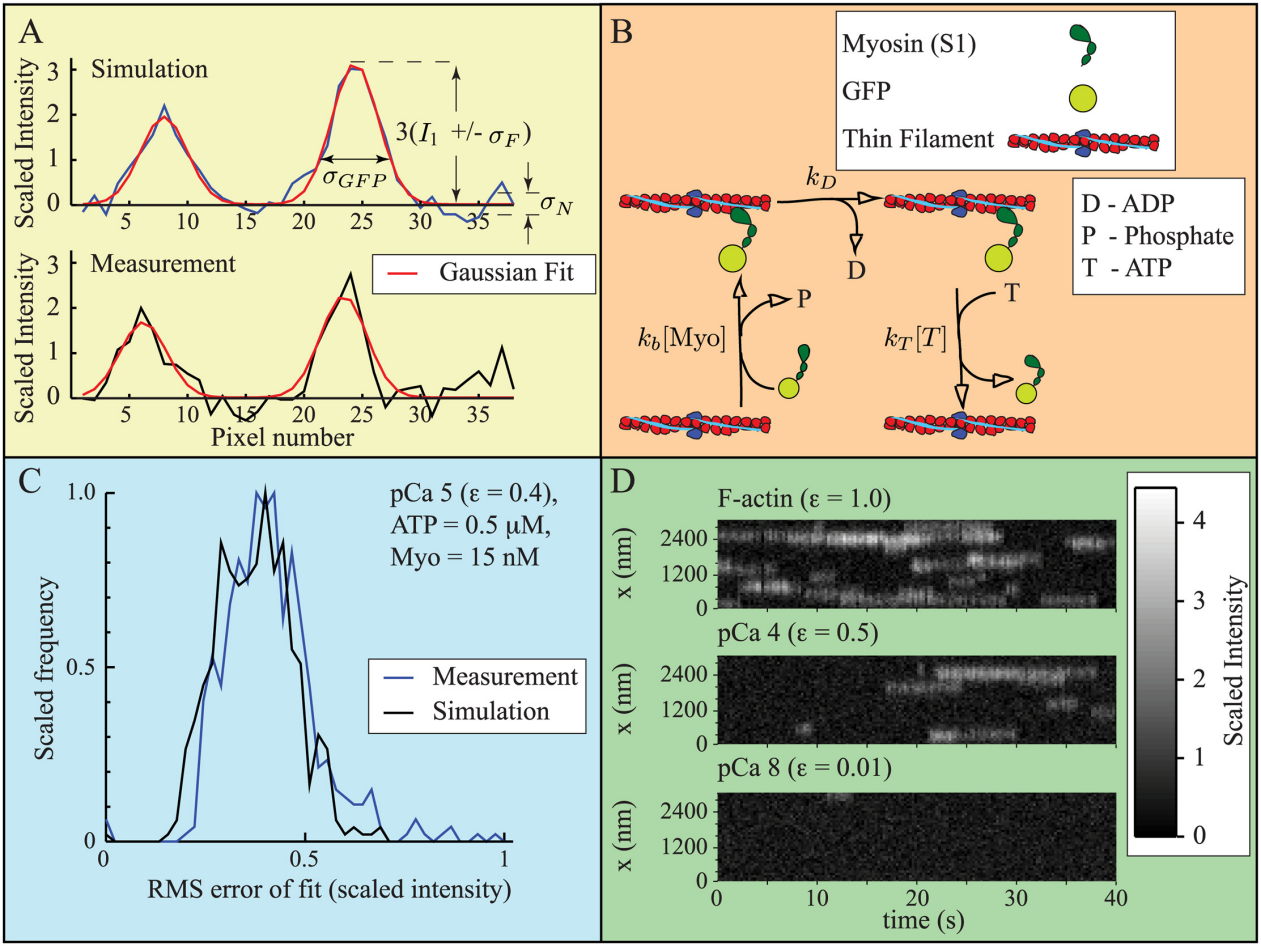
Parameter estimation and validation. A. Simulated data (top) reasonably reproduces experimental measurements (bottom), including noise. Simulations include two sources of noise: 1) uniform background noise with zero mean and standard deviation *σ*_*N*_; and 2) noise due to temporal fluctuations in fluorescent intensity, with zero mean and standard deviation *σ*_*F*_. The standard deviation of a diffraction limited spot, *σ*_*GFP*_ = 2 pixels, where one pixel is 80 nm. B. Kinetic scheme defining the interaction of a fluorescently-labeled myosin (GFP-S1) with an actin binding site. The scheme comes from [10], but has been modified to reflect the low myosin concentrations in the experiments [52]. C. Simulated and measured data generate similar fitting error, suggesting that we have successfully captured signal noise. The plot shows a histogram of root mean squared (RMS) error for each of 500 frames of simulated and measured data. D. By varying the calcium dependent fitting parameter *ε*, the model reasonably reproduces measurements at low and high calcium. At high calcium (pCa 4, where pCa is the negative log of the calcium concentration) Desai et al. [52], observed fewer binding events for thin filaments than for actin alone We can replicate this result with *ε* = 0.5 (top and middle). Binding was almost completely eliminated at very low calcium (pCa 8) [52], which we can replicate with *ε* = 0.01 (bottom). Note: for A and C, scaled intensity, defined in the text, gives a single GFP-S1 an intensity of 1. For D, zero is defined as the average minimum fluorescence achieved at each pixel. Here, scaled intensity is non-zero in the absence of GFP-S1, and a single GFP-S1 increases the signal by 1 unit.

In order to compare our model most directly to experimental measurements, we made the following assumptions:

1. A single excited GFP emits a constant average amount of light per unit time, *e*.
2. In the movies from Desai et al.[52], the recorded fluorescence intensity of a single GFP (*I*_1_) is inversely proportional to frame rate (*f*). Thus, a single GFP, imaged at *f* = 2 Hz, will be half as bright as a single GFP, imaged at *f* = 1 Hz.
3. There are two sources of signal noise. The first is background noise (e.g. electrical noise, unbound GFP-S1s, etc.) that is uniform across the image. This noise varies, depending on experimental condition. We assume this noise is Gaussian with mean zero and standard deviation *σ*_*N*_ (see Fig 3A).
4. The second source of signal noise is due to temporal variations in GFP intensity (e.g. flexing of the thin filament causing the center of the GFPs to deviate from the nominal average position of the thin filament, Fig 2A, red box). This noise is constant across all conditions. We assume this noise is Gaussian with mean zero and standard deviation *σ*_*F*_ (see Fig 3A).
5. Since GFP-S1s are coming out of solution, they can bind to either side of the actin filament. We assume each side is regulated independently [53]. Thus, each thin filament contains two independent systems of the sort shown in Fig 1C.
6. From fits to in vitro motility, we estimated C=11 [42]. Since $C=L_{C}/\Delta L$, where Δ*L* is the spacing between myosin molecules, this gives a value of *L*_*C*_ = 385nm, using Δ*L* = 35nm for myosin molecules attached to a glass surface [54]. Here, the GFP-S1s are not constrained by being bound to a surface so they can bind to each actin monomer, spaced 5.5 nm apart. Thus, Δ*L* = 5.5 nm, and so C=385/5.5=70. We assume that this value is independent of calcium concentration.
7. The value of *ε* is a function of calcium, and should lie between 0.003 (at very low calcium) and 1 (at high calcium, or in the absence of regulation) [42].
8. GFP-S1 myosin obeys the kinetic model described in Walcott et al. [10], but here it is coming out of solution and the rate limiting step is no longer the weak to strong transition, but rather the initial formation of the weak-binding complex (Fig 3B). Thus, the attachment rate per *μ*m of actin is $\kappa_{b}^{0}[\text{Myo}]$, where [Myo] is the concentration of GFP-S1 and $\kappa_{b}^{0}=0.2{\text{nM}}^{-1}\mu m^{-1}s^{-1}$ [52]. Since binding sites are spaced 5.5 nm apart along both sides of an actin filament, this corresponds to a binding rate of
  $$\begin{array}{ccc} k_{b}^{0} & = & \left(0.2{\text{nM}}^{-1}\mu m^{-1}s^{-1})(\frac{1\mu m}{1000\text{nm}})(\frac{5.5\text{nm}}{2\;\text{binding}\;\text{sites}}\right)\\ & = & 0.00055{\text{nM}}^{-1}s^{-1}\text{per}\;\text{binding}\;\text{site} \end{array}$$
9. Since myosin is coming out of solution, the myosin molecules do not apply forces on each other and so there is no mechanochemical coupling.

Assumptions 1 and 2, which posit that a single excited GFP emits, on average, a constant amount of light per unit time (*e*) and that the measured signal intensity (*I*(*t*)) is inversely proportional to frame rate (*f*), differ from the assumptions made by Desai et al. [52] that the recorded intensity of a GFP varies depending on experimental condition. We estimate *e* = 450 fluorescent units/s, giving a GFP intensity of *I*_1_ = 45 at *f* = 10 Hz and *I*_1_ = 118 at *f* = 3.8 Hz (see S1 Supplementary Material). To eliminate differences in measured signal intensity due to frame rate, we introduce a dimensionless quantity $I_{F}=I\left(t\right)f/e$, the scaled intensity. With this definition, a cluster of *n* GFP-S1s generates a scaled intensity of $I_{F}=n$.

To model the experiments, we estimated the background noise, defined by standard deviation *σ*_*N*_, and the variability in GFP intensity, defined by standard deviation *σ*_*F*_. For all simulations, we used *σ*_*F*_ = 0.22 (scaled intensity). The background noise varied depending on experimental condition, so we estimated *σ*_*N*_ for each condition. The values vary from *σ*_*N*_ = 0.21–0.36 (scaled intensity), and the exact values are given in Table 1. We can estimate overall signal noise by the root mean squared error between the measurement and the Gaussian fits. A comparison between the fitting error from a simulated experiment and from a measurement, consisting of 500 frames, suggests that our simulations have reasonably captured experimental noise (Fig 3C).

**Table 1:** Background noise used in simulations.

[Myo] (nM)	[ATP] (*μ*M)	pCa	*σ*_*N*_ (scaled intensity)
1	0.1	6	0.21
5	0.1	6	0.25
10	0.1	6	0.30
15	0.1	6	0.36
15	0.1	6	0.22
15	0.5	6	0.33
15	1	6	0.22
15	0.5	5	0.22
15	0.5	6	0.33
15	0.5	7	0.29

Details of parameter estimation in S1 Supplementary Material. Scaled intensity, defined in the text, gives a single GFP-S1 an intensity of 1.

With these assumptions, and given a value for the local coupling parameter *ε*, we can perform simulations of the experiments performed by Desai et al. [52]. To do so, we used the following algorithm:

- Step 1: Initiate a set of 546 sequentially ordered binding sites, representing one side of a thin filament with length 3*μ*m.
- Step 2: Use the Gillespie algorithm [41], and the rate constants and parameters given in Table 2, to determine when and where a GFP-S1 binds to one of these binding sites.
- Step 3: Update time and the state of that binding site to contain a bound myosin molecule.
- Step 4: Recalculate binding rate constants, *k*_*b*_, for each binding site according to the following equations for local coupling [42], which is Eq 1 with $k^{0}=k_{b}^{0}\left[\text{Myo}\right]$:
  $$k_{b}\left(i,j,C,\varepsilon \right)=\{\begin{array}{ccc} \varepsilon k_{b}^{0}\left[\text{Myo}\right] & : & i,j\geq C\\ \varepsilon^{i/C}k_{b}^{0}\left[\text{Myo}\right] & : & i<C,j\geq C\\ \varepsilon^{j/C}k_{b}^{0}\left[\text{Myo}\right] & : & i\geq C,j<C\\ \varepsilon^{(i+j-C)/C}k_{b}^{0}\left[\text{Myo}\right] & : & i,j<C,i+j>C\\ k_{b}^{0}\left[\text{Myo}\right] & : & i+j\leq C \end{array}$$ (2)
  where the integers *i* and *j* are the distance to the left and right, respectively, to the nearest occupied binding site.
- Step 5: Use the Gillespie algorithm [41] to determine when and where the next chemical reaction occurs, and update time and the state of each binding site.
- Step 6: Repeat steps 4 and 5 until time is greater than 35 seconds (this eliminates non steady-state effects).
- Step 7: Once time is greater than 35 s, start recording the state of the system at the appropriate frame rate (either 10Hz or 3.8Hz, depending on experiment).
- Step 8: Continue iterating the Gillespie algorithm (steps 4 and 5) until a sufficient number of frames has been generated (either 500 or 1000, depending on experiment).
- Step 9: Repeat steps 1–8, in order to simulate the other side of the thin filament.
- Step 10: Translate bound molecules into the appropriate fluorescent signal, including both variability in GFP intensity (*σ*_*F*_) and background noise (*σ*_*N*_). If a fluorescent molecule is bound for only a fraction of a frame, the fluorescent signal is assumed to be proportional to that fraction. That is, if frame rate were 10 Hz and a simulated molecule bound for 1/100th of a second (i.e. one tenth of the time the “camera” was collecting data), then a spot was generated that was 1/10th as bright as if a simulated molecule were bound for the entire time.

For each experimental condition, we performed five simulations.

**Table 2:** Model parameters.

Parameter	Value	Note
$k_{b}^{0}$	0.00055*nM* ^−1^ *s* ^−1^	From Desai et al. 2015
*k*_*D*_	350*s* ^−1^	From Walcott et al. 2012
*k*_*T*_	2*μM* ^−1^ *s* ^−1^	From Walcott et al. 2012
C	70	From Walcott 2013, adjusted for closer myosin spacing
*ε*, pCa 7	0.02	From fits
*ε*, pCa 6	0.06	From fits (see S1 Supplementary Material)
*ε*, pCa 5	0.4	From fits
*σ*_*F*_	0.22 (scaled intensity)	Estimated from Desai et al. 2015 (see S1 Supplementary Material)
*σ*_*N*_	0.21–0.36 (scaled intensity)	Estimated from Desai et al. 2015, see Table 1
*e*	450 fluorescent units/*s*	Estimated from Desai et al. 2015 (see S1 Supplementary Material)
*σ*_*GFP*_	160 nm (2 pixels)	From Desai et al. 2015

Note: scaled intensity, defined in the text, gives a single fluorescently-labeled myosin an intensity of 1.

To perform the simulations, we must specify the local coupling parameter *ε*. From assumption 7, we have 0.003 < *ε* < 1; we can further refine this estimate by comparison to measurements in the absence of regulation (*ε* = 1) and at pCa 4 and pCa 8 [52]. Even at high calcium (pCa 4), Desai et al. [52] observed a significant decrease in binding events in the presence of regulatory proteins compared to in their absence. We can reasonably replicate their data if we assume that 0.01 < *ε* < 0.5 in the presence of regulatory proteins, with the maximum and minimum values being obtained at pCa 4 and pCa 8, respectively (Fig 3D). This is consistent with the observation that, even at saturating calcium, myosin strong binding to actin induces a shift in the position of tropomyosin [55].

For each calcium concentration (pCa 5, 6 and 7), we estimated *ε* by trial-and-error, with the goal being to replicate the experimental measurements (see S1 Supplementary Material). Our model therefore has a single fitting parameter at each calcium concentration. The values used in our simulations are *ε* = 0.4 at pCa 5, *ε* = 0.06 at pCa 6 and *ε* = 0.02 at pCa 7. All model parameters are summarized in Table 2.

### Interpretation and Estimation of Coupling Parameters

In the model, local coupling is determined by the parameters *ε* and C. These affect the rate at which a myosin molecule binds to actin, *k*_*b*_, according to Eq 2. Importantly, the value of *k*_*b*_ depends on where neighboring myosin molecules are bound, thereby defining the local coupling in the system. Besides defining this reaction rate, the parameters *ε* and C also relate to mechanochemical properties of the thin filament. This connection is made explicitly, and mathematical expressions are provided, in Walcott 2013 (where the parameter *ε* is called *δ*) [42]; here we provide a more intuitive interpretation.

The parameter *ε* defines how the Tn-Tm beam decreases the attachment rate of an isolated myosin molecule. That is, if *ε* = 0.1, then the regulatory proteins decrease the binding rate of an isolated myosin 10-fold. Since calcium affects how easily myosin can bind to actin, *ε* is a function of calcium concentration. The value of *ε* can be related to physical properties of the thin filament, given the assumption that attachment rate depends exponentially on the energy required to displace the Tn-Tm beam, Δ*E*. In particular, since *ε* is defined as the reduction in an isolated myosin’s attachment rate due to the regulatory proteins, the exponential dependence of attachment rate implies that lim_*L*→∞_ exp(−Δ*E*) = *ε* (where Δ*E* is measured in *k*_*B*_ *T*, Boltzmann’s constant time temperature). Thus, Δ*E* asymptotes to −ln(*ε*) as *L* becomes large (Fig 1D).

The parameter C is defined as the ratio of two lengths: $C\equiv L_{C}/\Delta L$. The length Δ*L* is the spacing between neighboring myosin molecules interacting with a given actin filament, and depends only on how the myosin molecules are arranged in a given experiment. The length *L*_*C*_ is the critical separation between myosin molecules. If two myosin molecules are bound to actin and the separation between them is greater than *L*_*C*_, then they induce independent deformations of the Tn-Tm beam. That is, they are uncoupled. Conversely, if two myosin molecules are bound to actin and the separation between them is less than *L*_*C*_, then they completely activate the intervening length of thin filament.

The value of *L*_*C*_ depends on details of how the Tn-Tm beam interacts with myosin (*W*(*z*)), upon its mechanical properties (i.e. its flexural rigidity [42]), and upon the distance that the Tn-Tm beam is deformed upon myosin binding. These likely do not change upon changes in, say, ATP concentration or the geometry of the assay. Thus, we have assumed that *L*_*C*_ is constant between the experiments considered here and the motility assay [32, 42, 43]. In contrast, the interaction between the Tn-Tm beam and actin, *W*(*z*), might depend on calcium, and so it is possible that C varies with calcium. However, the local coupling model reasonably reproduces experimental measurements if C is assumed to be calcium independent, and only *ε* varies with calcium [32]. We therefore make that assumption here, fixing C (see Table 2) and allowing *ε* to vary in order to give the best fit between model and measurement.

## Results

**Fig 4:**
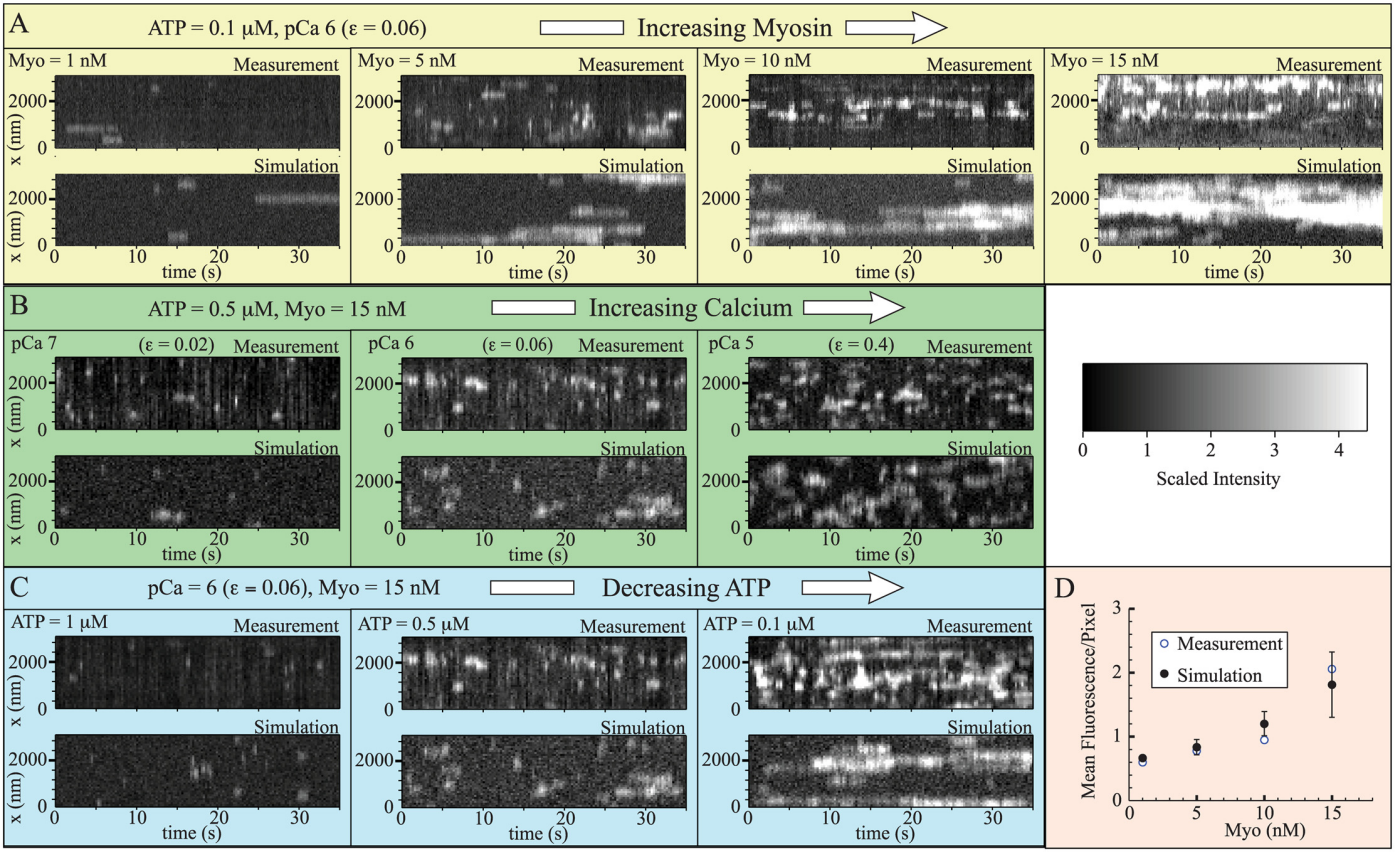
The model reproduces measured kymographs both qualitatively and quantitatively. In plots A-C, measurements [52] (replotted on a uniformly consistent scale) are at the top of each panel and simulations are at the bottom. The different experimental conditions are A. Increasing myosin (left to right), recorded at *f* = 10 Hz; B. Increasing calcium (left to right), recorded at *f* = 3.8 Hz; and C. Decreasing ATP (left to right), recorded at *f* = 3.8 Hz. D. Simulations reasonably capture mean fluorescence per pixel at variable myosin concentrations. For each myosin concentration, the mean scaled intensity of the measurements is shown, along with the mean and standard deviation of five simulations. The faster-than-linear increase in fluorescence with myosin indicates the presence of local coupling. Note: in all plots, zero is defined as the average minimum fluorescence achieved at each pixel. The scaled intensity, defined in the text, is non-zero in the absence of a myosin, and a single myosin increases the signal by 1 unit. The non-zero fluorescence in the absence of myosin, apparent in plot D, is a reflection of this offset.

We simulated nine different experimental conditions to follow Desai et al. [52], varying the amount of GFP-S1 ([Myo]), the amount of calcium, and the amount of ATP (pCa 6, ATP = 0.1*μ*M, [Myo] = 1, 5, 10, 15nM, 1000 frames collected at 10Hz; pCa 5, 6, 7, ATP = 0.5*μ*M, [Myo] = 15nM, 500 frames collected at 3.8 Hz; pCa 6, ATP = 0.1, 0.5, 1*μ*M, [Myo] = 15nM, 500 frames collected at 3.8Hz). Representative kymographs from measurements and simulation are shown in Fig 4. In all cases, agreement between simulation and experiments is reasonable.

We can make a more quantitative comparison between simulation and experiment by calculating the mean fluorescence per pixel in the kymographs. The agreement between simulation and experiments is again good (Fig 4D shows variable [Myo], the remaining plots are in the S1 Supplementary Material). Importantly, both the simulation and measurements show a non-linear dependence of mean fluorescence on [Myo]. Mean fluorescence (in units of scaled intensity) corresponds to binding probability and, since increasing [Myo] increases binding rate linearly, in the absence of local coupling mean fluorescence should increase, at most, linearly in [Myo]. The observation, in both experiment and simulation, that this curve has a faster-than-linear increase therefore implies the presence of local coupling in the experimental system and that the model accurately describes this local coupling.

**Fig 5:**
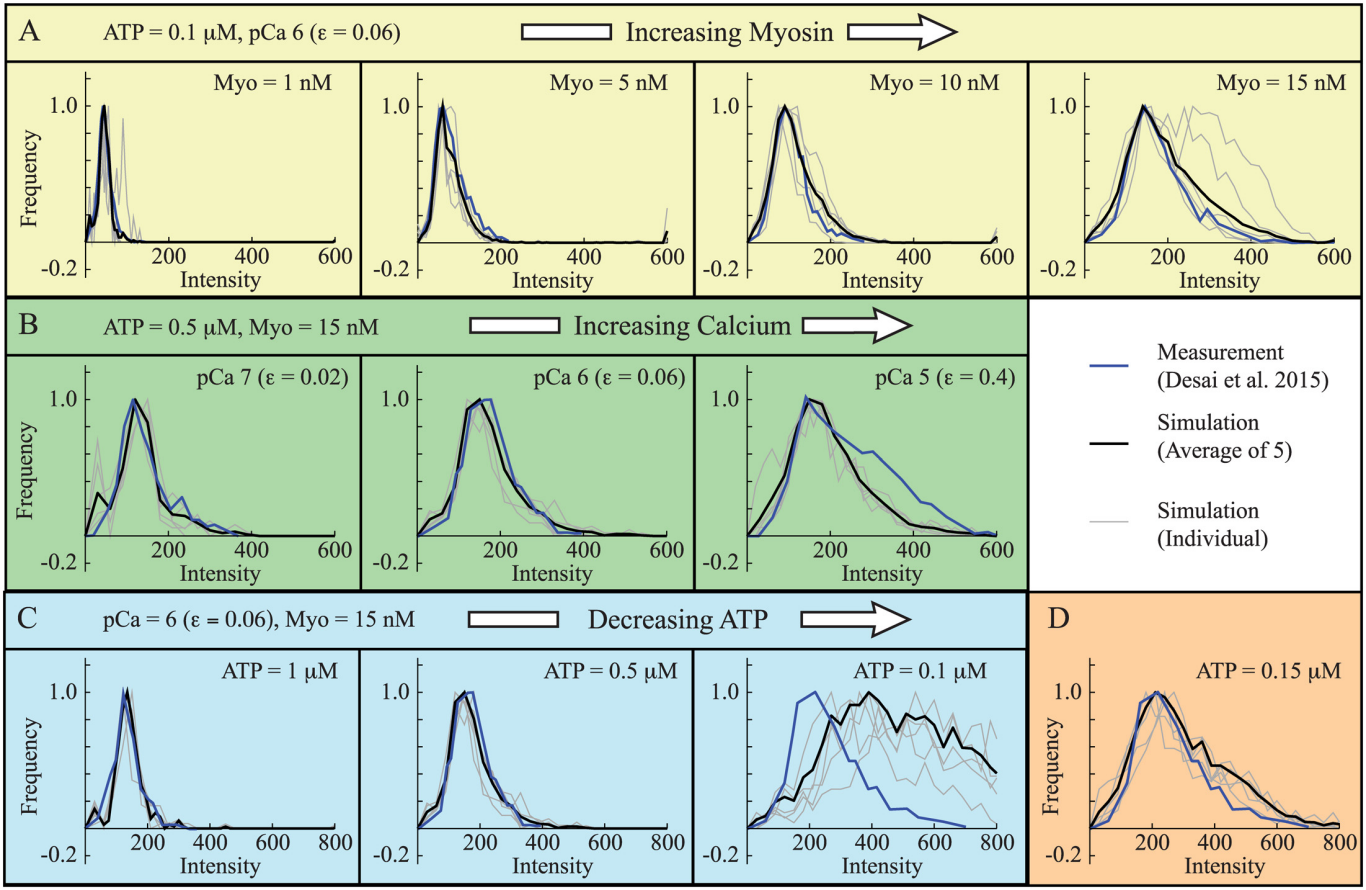
The model reproduces measured histograms. In all plots, histograms from measurements (blue) [52] are plotted along with histograms from five individual simulations (gray), and histograms of those five simulations pooled together (black). The different experimental conditions are A. Increasing myosin (left to right), 1000 frames recorded at *f* = 10 Hz; B. Increasing calcium (left to right), 500 frames recorded at *f* = 3.8 Hz; and C. Decreasing ATP (left to right), 500 frames recorded at *f* = 3.8 Hz. At each calcium concentration, there is only a single fitting parameter (*ε*). With the exception of the right panel in C, agreement between simulation and measurement is good. D. For the single case where model and simulation differ, we get good agreement between simulation and measurement with only a 50nM increase in ATP concentration. Differences between simulation and experiment are therefore likely due to the system’s sensitivity to parameters, so that small differences in experimental condition lead to large changes in measurement. Note: To compare simulations with measurements [52], all plots have units of intensity, which depends on frame rate. For plot A, collected at 10 Hz, the intensity of a single GFP-S1 is *I*_1_ = 45 intensity units. For plots B–D, collected at 3.8 Hz, the intensity of a single GFP-S1 is *I*_1_ = 118 intensity units.

The data contain more information than just mean fluorescence; each kymograph shows local clusters of fluorescence of varying brightness. The brightness of each spot can be quantified and plotted as a histogram, using custom code developed by Desai et al. [52]. We analyzed our simulated data with this code, as shown in Fig 2D, and the results are shown in Fig 5. With the exception of one measurement at pCa 6, [Myo] = 15 nM, and [ATP] = 0.1*μ*M (rightmost panel in Fig 5C), the agreement between simulation and measurement was good. Indeed, this agreement is remarkable since, at each calcium concentration, we had only one parameter (the binding probability: *ε*) with which to fit the data.

The reason that the simulations do not fit all of the experimental measurements becomes apparent upon closer inspection. There were two measurements performed at pCa 6, [Myo] = 15 nM, and [ATP] = 0.1*μ*M, one that was imaged at 10Hz (Fig 5A, rightmost panel) and one that was imaged at 3.8 Hz (Fig 5C, rightmost panel). While the simulations agree with the former, they do not agree with the latter. There are several possible reasons for this inconsistency, including variability in GFP fluorescence between the two experiments, but we believe the most likely explanation is that under these conditions where GFP-S1 binding is frequent and local coupling is strong, the system is highly sensitive to parameters. For example, with only a 50 nM increase in ATP concentration, the simulations are in reasonable agreement with the measurements (Fig 5D).

## Discussion

Accurate and efficient models of muscle contraction that are consistent with measurements across size scales could transform research in several fields. The study of genetic muscle defects, for example, would benefit from being able to predict how changes in molecular parameters due to mutation affect macroscale function [8, 56]. The study of human movement, and prosthetic design [57, 58], would benefit from being able to predict ATP hydrolysis in muscle as a function of muscle force and motion [59]. Most current muscle models are not able to achieve these tasks, in part, because they cannot accurately and efficiently describe the local coupling between myosin motors that occurs during activation. A recently proposed muscle model considers molecular mechanics and can be implemented using differential equations, thereby having the potential to describe this local coupling with unprecedented accuracy and efficiency [32, 42, 43]. However, until recent experimental advances [52], this model could only be tested against indirect measurements.

Here, we have tested this local coupling model [32, 42, 43] against these direct measurements of fluorescent myosin binding to thin filaments [52]. Not only does the model successfully describe myosin’s average binding probability, where we observe local coupling (Fig 4D), but it also describes the clustering of myosin molecules (Fig 5A–5C). Differences between measurement and simulation, seen in only one of the nine experiments, can be explained by the system being highly coupled and therefore sensitive to parameters (Fig 5D). This agreement between simulation and experiments suggests that the model captures the essential molecular events that underlie local coupling through the thin filament. While more work is necessary to understand how sarcomeres are coupled and the role of accessory proteins, this success at the molecular scale, in conjunction with the success of the model at the ensemble and fiber scale under more physiologically realistic conditions [32, 42, 43], represents progress in understanding the role of local coupling in muscle contraction and, more broadly, a multi-scale understanding of muscle contraction.

Although the simulations and measurements agree, the model requires only a single myosin head to activate the thin filament; however, Desai et al [52] inferred that two heads are required. This difference is explained by alternate assumptions. Here, we assume that an excited GFP emits the same amount of light per unit time (*e*), regardless of condition, and that the intensity of a GFP recorded by the camera is inversely proportional to frame rate (*I*_1_ = *e*/*f*). Desai et al. [52] did not explicitly define the fluorescence of single GFPs (*I*_1_); instead, the intensity distributions (see Fig 2D) were fit with a series of Gaussians of fixed standard deviation (see S1 Supplementary Material for details of this method). Further experimental measurements are necessary to ultimately resolve which assumption is correct and determine whether or not a single myosin can activate a thin filament (see the Proposed experiments section); but below we present evidence in favor of constant GFP emission.

### Evidence for Constant GFP Emission

**Fig 6:**
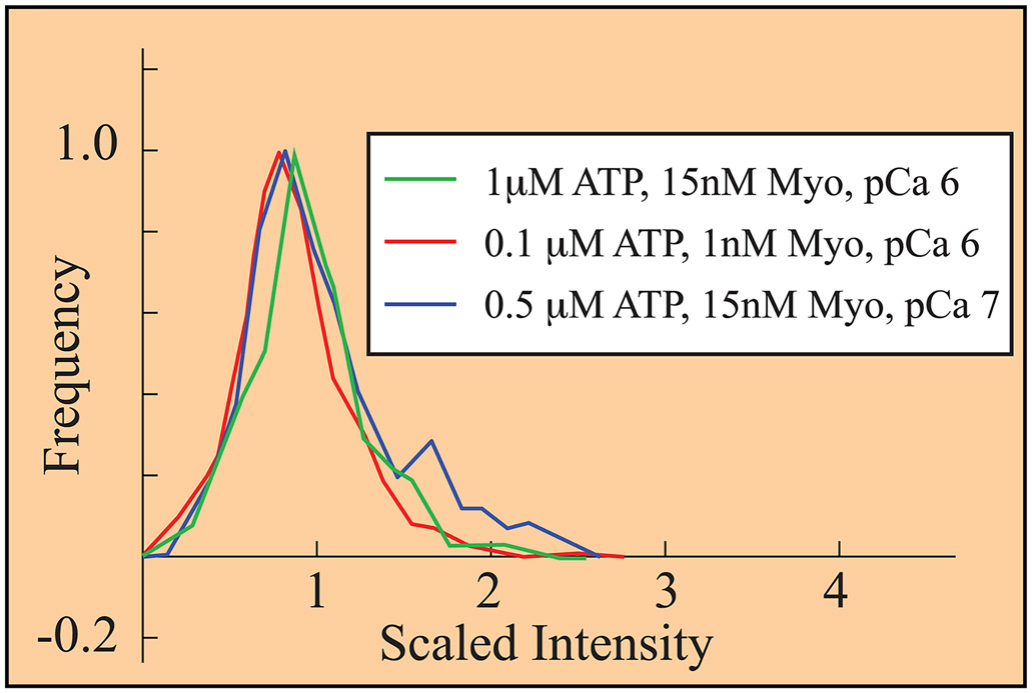
Histograms, collected under three different experimental conditions [52], collapse upon rescaling fluorescence. Scaled intensity, defined in the text, gives a single fluorescently-tagged myosin (GFP-S1) an intensity of 1. The observation that each histogram has a peak near 1 suggests that 1) under these three conditions, mostly single GFP-S1s bind; and 2) the emission of an excited GFP is constant and measured fluorescence is inversely proportional to frame rate. This latter is a central assumption of our analysis.

To support the assumption of constant GFP-S1 fluorescence intensity, at *e* = 450 fluorescent units/s, we reanalyzed the data from Desai et al [52]. If GFP-S1 fluorescence varied significantly, then the fluorescent spots from single GFP-S1s between conditions could not be rationally rescaled. Using $I_{F}=If/e$ we rescaled the histograms of fluorescent intensity observed by Desai et al. [52] under three conditions where isolated single GFP-S1s are expected to dominate binding. These conditions are: high ATP (pCa 6, ATP = 1*μ*M, [Myo] = 15nM, *f* = 3.8Hz), low myosin (pCa 6, ATP = 0.1*μ*M, [Myo] = 1nM, *f* = 10Hz), and low calcium (pCa 7, ATP = 0.5*μ*M, [Myo] = 15nM, *f* = 3.8Hz). The resulting histograms are similar, supporting the assumption of equal intensity (Fig 6).

### Proposed Experiments

From this study it is clear that the local coupling model agrees well with data recently collected using single molecule fluorescence. The wider agreement of this model with bulk and physiological data suggests this model has the potential to describe and predict systems behavior at multiple levels. Further refinement of this model provided by single molecule data requires challenging one outcome from the experiments: that two heads are required to activate the thin filament. To experimentally validate or refute this outcome, the transition from one to two bound molecules must be observed. However, Desai et al. [52] used a steady-state approach to analyze their data, leaving the time-domain unexplored. Determining if two heads are required for activation can be most effectively done using fast imaging techniques with high spatial resolution. Such experiments can directly determine if the binding of a second head is required to generate activation. This would manifest as a change in the apparent second order binding rate constant once two heads are bound, with no (or little) change if a single head is bound. As an additional benefit, these measurements could also define model parameters. For example, determining the spatial separation between the first two heads to bind and the degree of collaboration provides a direct measure of *L*_*C*_, the critical length over which two myosin molecules communicate.

Desai et al’s [52] conclusion that few isolated GFP-S1s are bound to an activated thin filament not only requires that binding be coordinated, but also requires that unbinding be coordinated. In support of this view, they do not clearly observe stepwise dissociation [52]. The model described in this study predicts that unbinding should occur stepwise and that single GFP-S1s should exist in activated conditions. Coordinated detachment requires that the regulatory proteins act to remove low numbers of myosin molecules, which would challenge the view that regulation affects only myosin’s attachment rate [11]. Again, high spatial and temporal imaging in these challenging conditions would provide a measurement of the process of thin filament relaxation—a process of high relevance to disease [60].

### Conclusions

Recent experimental observations [52] of thin filament activation by single myosins were well predicted by a local coupling model described in this study. The importance of this result is that a relatively simple model of local coupling, which can be incorporated into efficient differential equation models, now fits in vitro motility data at high and low calcium [42], in the presence of MyBP-C [43], fiber data [32] and, as we have just shown, direct molecular-scale measurements. This validation of the model across size scales suggests that it captures the essential phenomenology of the local coupling between myosin molecules that occurs upon muscle activation. As it captures this coupling across scales, the model has the potential to provide insight into precisely how molecular level parameters affect macroscopic function—a particularly important problem, since mutations that affect local coupling have been implicated in some genetic cardiomyopathies [61].

## Supporting Information

S1 Supplementary Material Additional details of parameter estimation, further comparison of the model with the data, and details of data analysis. (PDF)
