# Supplementary material for: Direct Measurements of Local Coupling between Myosin Molecules Are Consistent with a Model of Muscle Activation
Source: PLoS Comput Biol. 2015 Nov 4;11(11):e1004599. doi: 10.1371/journal.pcbi.1004599 (PMC4633106; doi:10.1371/journal.pcbi.1004599)
Supplement: S1 Supplementary Material — (PDF) [file pcbi.1004599.s001.pdf]

Supplementary material to accompany "Direct measurements of local coupling between myosin molecules are consistent with a model of muscle activation."

Sam Walcott and Neil Kad

August 9, 2015

We compared a model of local coupling between myosin molecules [1, 2, 3] to direct observations of this coupling [4]. Although the model agreed with the measurements, there is a discrepancy between the two. Desai et al. [4] conclude that two myosin heads are required to activate the thin filament, while the model assumes that a single myosin head can activate it [1, 2, 3]. Here, we discuss parameter estimation in the model (Section 1), present plots of average fluorescent intensity for the simulations and experiments (Section 2), and discuss some details of the analysis, performed by Desai et al. [4], that led them to conclude that two myosin heads are required to activate the thin filament (Section 3).

1 Parameter estimation

In order to model the measurements of Desai et al. [4], we had to estimate four parameters: σ_N the standard deviation of the assumed Gaussian background noise; σ_F the standard deviation of the assumed Gaussian temporal fluctuation in GFP intensity; e the fluorescent emission of a GFP; and ε which determines the degree of regulation. All parameters, except ε, were estimated prior to fitting the data with the model. We varied ε in order to optimize the fit of the model to the data.

We estimated the background noise, σ_N, individually for each experimental condition. The values used in the model are listed in Table 1 of the main text. To estimate σ_N from a kymograph, we first removed spatial fluctuations in intensity. To do so, we calculated the minimum fluorescence at every pixel along the actin filament. We then fit a fifth-order polynomial to this minimum fluorescence as a function of position along actin. The resulting curve defined zero fluorescence for every pixel along actin.

We then plotted a histogram of the fluorescence of every pixel in a kymograph. The data show a peak, which corresponds to the background noise, and an extended tail toward positive fluorescence, which corresponds to the binding of fluorescent myosin (GFP-S1). We estimated the standard deviation of the histogram by matching a Gaussian to the distribution to the left of the mean, thereby only fitting the background noise (see Fig. 1A).

To estimate σ_F and e, we analyzed a histogram, which plots the frequency of spots of a given intensity, measured under conditions where we expected to see primarily single molecules binding (pCa 6, [ATP] = 0.1 μM, [Myo] = 1 nM). The maximum of this peak was around $I_1 = e/f = 45$ intensity units (Fig. 1B). Given that these data were collected at a frame rate of $f = 10$ Hz, this gives a value of $e = fI_1 = 450\text{s}^{-1}$.

Since the histogram is constructed by fitting the raw data with a Gaussian, we expect that the contribution of σ_N to the signal is small. One might then expect the histogram to be Gaussian with standard deviation σ_F, but the histogram is not exactly Gaussian. This is likely due to imperfections in the fitting algorithm used to construct the histogram. However, the histogram is approximately Gaussian near the peak, so we estimated $\sigma_F = 0.22$ from matching a Gaussian to this peak. In support of the view that the non-Gaussian shape of the histogram is due to the fitting algorithm, and that the width of the peak can be reasonably used to estimate σ_N, simulations with only single binding events generate histograms that are similar to observations (Fig. 1B).

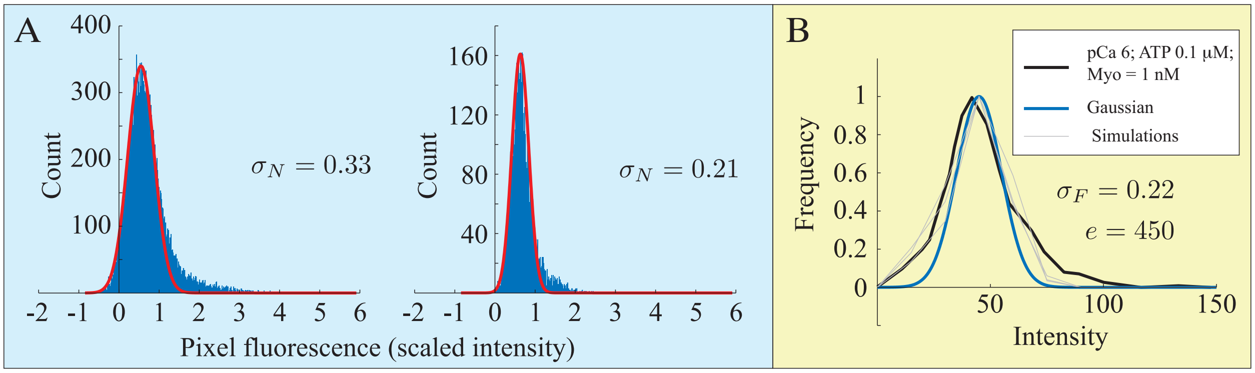

Figure 1: Estimating noise, σ_N and σ_F, and fluorescence emission, e, from the measurements of Desai et al. (2015). A. Estimating the background noise from kymographs. On the left is pCa 6, [ATP] $= 0.5\mu$M, [Myo] $=$ 15 nM (collected at a frame rate $f = 3.8$ Hz); on the right is pCa 6, [ATP] $= 0.1\mu$M, [Myo] $= 1$ nM (collected at $f = 10$ Hz). The histograms show the fluorescent intensity (in units of scaled intensity) for every pixel in a kymograph. Gaussians (red) match the left side of the distribution, with standard deviations $\sigma_N = 0.33$ (left) and $\sigma_N = 0.21$ (right), both in units of scaled intensity. The deviation from the Gaussian on the right side of the distribution is due to the binding of fluorescent myosin. B. Estimating the fluorescence emission, e, and temporal fluctuations in fluorescent intensity, σ_F. Assuming most binding events are from single myosin molecules when myosin concentration is low (pCa 6, [ATP] $= 0.1\mu$M, [Myo] $= 1$ nM), a histogram of the intensity of every spot in a kymograph should have a peak at the fluorescence of a single molecule, $I_1 = e/f$. The peak occurs at an intensity of 45, giving $e = 450\text{s}^-1$, since the data were collected at $f = 10$ Hz. The spread in the histogram near the peak is due to temporal fluctuations in intensity, and is well-fit by a Gaussian with standard deviation $\sigma_F = 0.22$ (blue), in units of scaled intensity. Deviations from the Gaussian are likely due to due to the algorithm used to determine the fluorescent intensity of a spot, since simulations with only single binding events generate histograms that are similar to observations (gray).

To estimate ε, we fit the data [4]. A series of preliminary simulations suggested that $\varepsilon \approx 0.06$ fit the data at pCa 6 the best. To quantify this, we performed simulations of the experiments at variable [Myo] (pCa 6, ATP $= 0.1\mu$M, [Myo] $= 1, 5, 10, 15$nM, 1000 frames collected at 10Hz), with $\varepsilon = 0.04$, 0.06, and 0.08. We compared both the distributions of clusters (Fig. 2A), and mean fluorescence per pixel (Fig. 2B). In all cases, $\varepsilon = 0.06$ was the best (Fig. 2C). We therefore used this value for pCa 6. Since there was only a single measurement at pCa 5 and pCa 7, we did not perform as detailed an analysis, but rather estimated $\varepsilon = 0.01$ and $\varepsilon = 0.4$, respectively, from preliminary simulations.

2 Average fluorescence

In the main text, the comparison between model and data is presented qualitatively in Figure 4A-C, where simulated and measured kymographs are displayed side-by-side. To obtain a more quantitative comparison, we determined the average fluorescence per pixel in each kymograph. In the main text, we present only measurements at variable [Myo] (Fig. 4D). All measurements are shown in Fig. 3. The agreement between simulation and measurement is good.

3 More details of constant vs. variable GFP emission

3.1 Desai et al's analysis

The model of local coupling between myosin molecules [1, 2, 3] assumes that a single myosin can activate the thin filament; Desai et al [4] conclude that two myosin heads are required to activate the thin filament. Desai

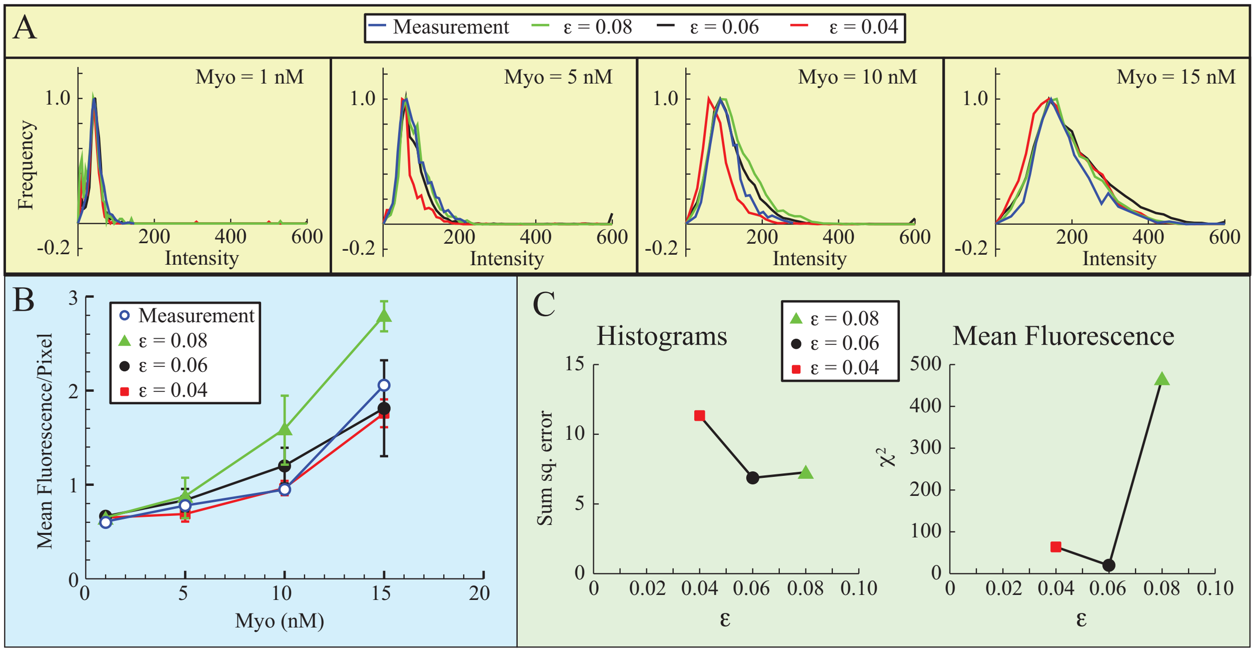

Figure 2: Estimating ε at pCa 6. A. Comparing simulations with $\varepsilon = 0.04$ (red), 0.06 (black) and 0.08 (green) to measured histograms (blue) from Desai et al. [4]. B. Comparing simulations with $\varepsilon = 0.04$ (red), 0.06 (black) and 0.08 (green) to the average pixel fluorescence (in scaled intensity) in kymographs (blue) from Desai et al. [4]. C. A quantitative comparison of the error in A and B shows that $\varepsilon = 0.06$ gives the best agreement with the data. For B, scaled intensity, defined in the main text, gives a single GFP-S1 an intensity of 1

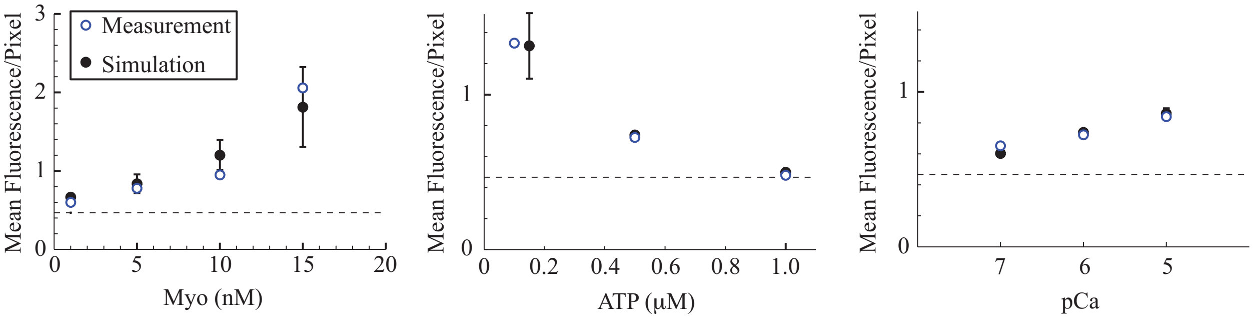

Figure 3: Plots of average pixel fluorescence for kymographs measured under various conditions. The left plot, at variable myosin, can be found in the main text as Fig. 4D. The other two plots show variable ATP and variable calcium. The model (solid dots, SD error) reasonably agrees with the data from Desai et al. [4] (hollow dots). Note that the lowest ATP measurement (middle plot) was simulated at [ATP]=0.15 μM, as discussed in the main text. For each pixel, zero fluorescence is defined as the minimum value it achieves during the kymograph. In each plot, the apparent background fluorescence is indicated as a dashed line. Fluorescence is measured in scaled intensity, defined in the text, which is non-zero in the absence of a myosin, and a single myosin increases the signal by 1 unit.

et al's [4] conclusion follows from their analysis of the histograms they measured (see Fig. 2 of the main text for the construction of these histograms, and Fig. 5 of the main text for the histograms themselves). We now summarize this analysis.

Each measured histogram contains the fluorescent intensity of every spot in a 500 or 1000 frame movie. Each fluorescent spot comes from a cluster of GFP-tagged myosin molecules (GFP-S1s). Given that

1. each GFP-S1 has an average fluorescent intensity I_1
2. a cluster of i GFP-S1s has a measured intensity of $iI_1 \pm \sigma_S$, where σ_S represents signal noise
3. signal noise is Gaussian and its magnitude is independent of the number of GFP-S1s in the cluster

then each histogram $F(I)$ arises from the following sum

$$F(I) = \sum_{i=1}^{\infty} a_i \exp\left(\frac{(I - iI_1)^2}{2\sigma_S^2}\right) \tag{1}$$

The coefficients, a_i, determine the relative frequency of clusters of i molecules in the histogram. Thus, for example, if a histogram consists only of clusters of single GFP-S1s, then $a_1 = 1$ and $a_2 = a_3 = a_4 = \cdots = 0$.

Desai et al [4] determined the coefficients, a_i, by first measuring σ_N for an isolated GFP and then fitting their histogram with the following equation

$$F(I) = \sum_{i=1}^{\infty} a_i \exp\left(\frac{(I - I_i)^2}{2\sigma_N^2}\right) \tag{2}$$

allowing the a_i and I_i's to vary in order to optimize the fit (Fig. 4A). Each time they performed this fit, they found that the optimal I_i values occurred at regularly spaced intervals – consistent with Eq. 1, where $I_i = iI_1$. Thus, one might expect that the lowest I_i is I_1, corresponding to a cluster of one GFP-S1, the next $2I_1$, corresponding to a cluster of two GFP-S1s, and so on. If so, then if the I_i's are sorted from small to large, and plotted as a function of apparent cluster size (i.e. 1 for the first, 2 for the second, and so on), the resulting curve should be linear with slope I_1 and should extrapolate to 0 at a cluster size of 0. Although linear curves were always observed, frequently the line did not extrapolate to 0 (Fig. 4B, black curve). Instead, the curve extrapolated to 0 only upon assuming that the first cluster contained two GFP-S1s, the second three, and so on (Fig. 4B, red curve). Based on this observation, Desai et al [4] conclude that single GFP-S1s cannot bind to the thin filament; rather, two or more GFP-S1s are required to activate the thin filament.

3.2 Analysis of simulated data

Given that the model, which assumes a single GFP-S1 can activate the thin filament, successfully reproduces the measurements, one might guess that the measured histograms do not necessarily imply that two or more GFP-S1s are required to activate the thin filament. However, there are subtle differences between the measured and simulated histograms. It is therefore possible that the analysis of Desai et al [4] is sensitive to these subtle differences, so that the simulated data, when analyzed, will differ from the analysis of the measurements. We therefore performed Desai et al's [4] analysis on the simulated measurements with variable [Myo] (Fig. 4).

We used a slightly different fitting algorithm than Desai et al [4]. Our algorithm is as follows:

1. Use linear interpolation to represent a given histogram with 1000 equally spaced points between 0 and 600.
2. Determine the intensity (I_{max}) at which the maximum frequency (F_{max}) occurs.
3. Use a non-linear optimization algorithm (Matlab's fminsearch function), starting from an initial guess of mean I_{max} and amplitude F_{max}, to fit the histogram with a single Gaussian, with standard deviation σ_N, allowing its mean and amplitude to vary to minimize the least squares error between the Gaussian and the interpolated histogram
4. Determine the intensity (I_E) at which the measurement and fit have a maximum difference (F_E).

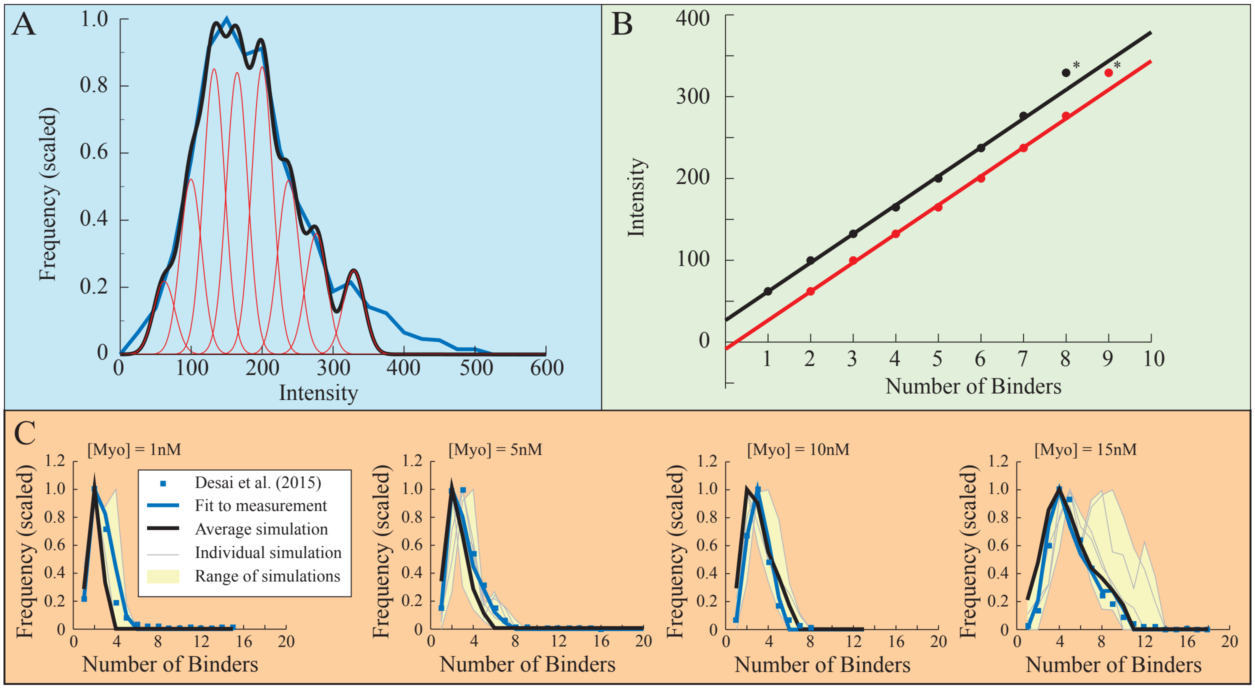

Figure 4: Desai et al's analysis, when applied to simulated or measured histograms, gives similar results. A. In the first step of the analysis, a histogram is fit with Gaussians of fixed standard deviation. Here, a simulated histogram (pCa 6, ATP = 0.1μM, [Myo] = 15nM, 1000 frames collected at 10Hz) is fit with eight Gaussians, each of standard deviation $\sigma = 15$. B. In the second step of the analysis, the Gaussians are ordered, numbered sequentially and plotted. In all cases, a linear curve results. Sometimes, that linear curve does not pass through the origin (black), but when the first point is assigned to two molecules, the linear curve passes through the origin (red). Note: the starred point is considered an outlier and is not included in the linear fit. C. In the third step of the analysis, the amplitudes of the Gaussians that correspond to each number of binders are plotted. We wrote our own algorithm for this analysis, which differs slightly from Desai et al's [4] algorithm. We used our algorithm to analyze measured histograms (blue), and simulated histograms (gray, individual, black, average, yellow, range) collected at 10Hz, variable myosin, pCa 6, ATP = 0.1μM, 1000 frames. Analysis of the measurements and simulations with our algorithm yields similar results to those reported in Desai et al. [4] (blue squares).

5. Use a non-linear optimization algorithm (Matlab's fminsearch function), starting from an initial guess of the previous best-fit with one additional Gaussian, with standard deviation σ_N, of mean I_E and amplitude F_E.

6. Repeat steps 4 and 5 until no improvement occurs, and/or two Gaussians with similar means are observed.

To validate this algorithm, we analyzed the measured histograms at variable [Myo], allowing σ_N to vary in order to optimize the fit to Desai et al's [4] analysis of these histograms. The results, shown in Fig. 4C with Desai et al's [4] analysis as blue squares and our fit as blue lines, are in reasonable agreement. Further, the best-fit values of $\sigma_N = 9, 12, 17$ and 15 at [Myo] = 1, 5, 10 and 15 nM, respectively, are in reasonable agreement with our estimates (see Parameter Estimation in Supplementary Material) of $\sigma_N = 9.5, 11.25, 13.5$ and 16.2 at [Myo] = 1, 5, 10 and 15 nM, respectively.

When we used this algorithm to analyze the simulated data, the results are similar to those from the measurements (Fig. 4C). Further, many of the fits exhibited the same non-zero extrapolation reported in Desai et al [4] (e.g. Fig. 4B is from a simulation at [Myo] = 15 nM). While these results are the basis for the

conclusion that two or more myosin molecules are required to activate the thin filament, these simulations assume that a single myosin can activate the thin filament.

References

[1] Walcott S. A differential equation model for tropomyosin-induced myosin cooperativity describes myosinmyosin interactions at low calcium. Cell Mol Bioeng. 2013;6: 13-25.

[2] Walcott S. Muscle activation described with a differential equation model for large ensembles of locally coupled molecular motors. Phys Rev E. 2014;90: 042717.

[3] Walcott S, Docken S, and Harris SP. Effects of cardiac myosin binding protein-C on actin motility are explained with a drag-activation-competition model. Biophys J. 2015;108: 10-13.

[4] Desai R, Geeves MA, and Kad NM. Using fluorescent myosin to directly visualize cooperative activation of thin filaments. J Biol Chem. 2015;290: 1915-1925.
